# Association of Blood Pressure With Stroke Risk, Stratified by Age and Stroke Type, in a Low-Income Population in China: A 27-Year Prospective Cohort Study

**DOI:** 10.3389/fneur.2019.00564

**Published:** 2019-05-29

**Authors:** Xin Du, Conglin Wang, Jingxian Ni, Hongfei Gu, Jie Liu, Jing Pan, Jun Tu, Jinghua Wang, Qing Yang, Xianjia Ning

**Affiliations:** ^1^Department of Cardiology, Tianjin Medical University General Hospital, Tianjin, China; ^2^Department of Geriatrics, Tianjin Medical University General Hospital, Tianjin, China; ^3^Department of Neurology, Tianjin Medical University General Hospital, Tianjin, China; ^4^Laboratory of Epidemiology, Tianjin Neurological Institute, Tianjin, China; ^5^Key Laboratory of Post-Neuroinjury Neuro-repair and Regeneration in Central Nervous System, Tianjin Neurological Institute, Ministry of Education, Tianjin, China; ^6^Department of Neurology, Tianjin Haibin People's Hospital, Tianjin, China

**Keywords:** stroke, blood pressure, epidemiology, cohort study, risk factors

## Abstract

Association of stroke risk with new blood pressure criterion 2017 is unknown in China. We assessed the association between blood pressure (BP) values and stroke risk in a low-income population in Tianjin, China. Systolic blood pressure (SBP) and diastolic blood pressure (DBP) values were categorized into five strata and strokes were recorded as stroke, ischemic stroke, and hemorrhagic stroke. Stroke risk was analyzed according to blood pressure stratum using Cox regression analysis. Overall, 4,017 residents (age, ≥18 years) were included in this prospective cohort study. Over a 27-year follow-up period (total, 86,515.78 person-years), 638 participants experienced first-ever strokes. The stroke risk was higher among individuals with SBPs ≥140 mmHg or DBPs ≥90 mmHg than among those with SBPs < 130 mmHg or DBPs < 80 mmHg (reference group), after adjusting for covariates. However, hemorrhagic stroke risk increased only in participants with SBPs ≥160 mmHg. The stroke risk increased for individuals < 65-years-old having BP values ≥130/80 mmHg and for individuals ≥65-years-old with BP values ≥160/90 mmHg. To reduce the stroke burden in China, target BP goals must be established for adults, with different targets for the middle-aged and the elderly segments of the population. These results are very important for guiding clinical practice and may be generalized to other developing countries experiencing rapid economic development and where transitions in the spectrum of prevalent diseases have occurred.

## Introduction

Globally, elevated blood pressure (BP) is the strongest modifiable risk factor for cardiovascular disease (CVD) and related disabilities; CVD caused almost 10 million deaths, worldwide, in 2013 (1). In China, in 2013, CVD (primarily stroke and ischemic heart disease) accounted for 2.5 million deaths (28% of overall deaths) and 15% of total disability-adjusted life-years (1, 2). Currently, ~28% of Chinese adults have hypertension, and its prevalence has increased significantly during recent decades. Moreover, the rates of hypertension awareness, treatment, and control remain extremely low, suggestive of a substantial and unnecessary future disease burden (3).

China is an agricultural country, with approximately half of the population living in underdeveloped rural areas where the prevalence of hypertension is high and treatment and control rates are low. Our previous study showed that the age-standardized prevalence of hypertension, among rural residents 35–74-years-old, was 51.7% in 2011, but its treatment (43.5%) and control (12%) rates were poor (4). Furthermore, the incidence of first-ever strokes in this population has steadily increased over time. For example, between 1992 and 2012, the annual increase in the overall incidence of first-ever stroke was 6.5% (5).

Over the past few decades, BP-related CVD, especially stroke, has become a major public health challenge (6, 7). Many studies have shown that stroke is the predominant CVD, in China (6, 8–11). Graded associations between elevated systolic (SBP) and diastolic (DBP) BP levels and increased CVD risk have been demonstrated in previous observational studies (4, 12). Specifically, the risk of CVD increases in a log-linear fashion between SBP levels of < 115 mmHg to >180 mmHg and between DBP levels of < 75 mmHg to >105 mmHg (12). Further, baseline BP measurements are stronger predictors of long-term CVD or stroke events than are other conventional risk factors (13–15). However, the association of BP levels with the risk of different stroke types, by age, is unknown in China, especially among low-income individuals. Thus, we used a 27-year study to assess the relative stroke risk associated with different BP levels in a low-income population, in China; the risk was assessed according to patient age, sex, and stroke type.

## Materials and Methods

### Study Population

This is a population-based cohort study, which conducted in 1991. The study population involved individuals participating in the Tianjin Brain Study, a previously described, population-based, stroke surveillance study that began in 1985 in a township in Tianjin, China (4, 16–18). Briefly, the study population resided in 18 administrative villages in Yangjinzhuang, a township in Tianjin; 95% of the residents were low-income farmers. The primary source of income was grain production, and the annual per capita income was < 100 USD in 1991 and < 1,000 USD in 2010 (19).

### Sampling Method

The sampling method used in this cohort study was reported previously (4). Briefly, we grouped the villages, according to geographical locations, as east, south, and north. We randomly sampled two villages from each location, using a stratified cluster sampling method, selecting all residents ≥15-years-old without a history of CVD or stroke. For this study, only participants ≥18-years-old were included to determine the association of BP with stroke risk.

The study protocol was approved by the ethics committee of Tianjin Medical University General Hospital; written informed consent was obtained from each individual.

### Baseline Information

Individual demographic characteristics (including sex, age, and educational attainment), self-reported disease history (including hypertension, diabetes, stroke, and CVD), and lifestyle factors (including smoking and alcohol consumption status and physical activity) were collected. All information was collected by local, trained research staff who conducted face-to-face interviews; the interviews also included physical examinations to determine BP, height, and body weight.

### BP Measurement

BP was measured at baseline as described previously (4). Briefly, standardized BP measurements were performed using a mercury sphygmomanometer with the cuff size adjusted to the individual's arm circumference. The cuff was placed on the arm at the level of the heart, and the BP was recorded as the mean of two measurements, 5 min apart, with the participant resting in the supine position; the SBP and DBP values were determined according to Korotkoff sounds I and V. If the difference between the two readings was not within 10 mmHg (SBP) and/or 5 mmHg (DBP) or if the measurement reached the criteria for hypertension, further two readings were obtained after the participant rested for an additional 20 min.

### Risk Factor Definitions

Hypertension was defined as a self-reported history of hypertension, current antihypertensive medication(s) use, or a baseline SBP/DBP >140/90 mmHg. Diabetes, stroke, and CVD determinations were based on self-reported disease histories. The body mass index (BMI) was calculated as the weight (kg) divided by the square of the height (m); BMIs were used to define normal weight (BMI < 24 kg/m^2^), overweight (BMI = 24–27.9 kg/m^2^), and obesity (BMI ≥28 kg/m^2^) (20). Physical activity was defined as physical activity for ≥30 min/day, at least 5 days/week.

### Stroke and Type Diagnosis

Stroke was defined as an acute-onset focal neurological deficit of vascular etiology persisting for >24 h, including both ischemic and hemorrhagic stroke subtypes. Hemorrhagic stroke was defined as an intracerebral hemorrhage (ICH) or a subarachnoid hemorrhage, and ischemic stroke was defined as a thrombotic brain infarction, cardioembolic stroke, or lacunar infarct; an undetermined stroke was defined as a stroke that could not be classified into either broad subtype. Stroke patients were only diagnosed if they demonstrated symptomatic strokes, with significant clinical symptoms and signs. Transient ischemic attacks and silent strokes (diagnosed by imaging only) were excluded, but stroke cases with histories of transient ischemic attacks were regarded as incident events. Patients demonstrating transient symptoms and having concurrent neuroimaging evidence of brain infarctions were considered as stroke cases, based on the “tissue” definition (21). In the early phase of this study (1992–1998), the events were confirmed primarily based on clinical examinations by senior neurologists for non-hospitalized patients and using medical records for hospitalized patients.

### Statistical Analysis

Continuous variables (age, BP, and BMI) are presented as means and standard deviations (SDs); categorical variables are presented as frequencies with 95% confidence intervals (CIs). Age-standardized incidences were calculated using the direct method and world-standard population age groups: < 35, 35–39, 40–44, 45–49, 50–54, 55–59, 60–64, 65–69, 70–74, and ≥75 years (22). Subgroup analyses were conducted to evaluate the first-ever stroke risk by age group (18–34 years, 35–44 years, 45–54 years, 55–64 years, 65–74 years, and ≥75 years), education level (illiterate, 1–6 years, 7–9 years, and ≥10 years of education), SBP group (< 130, 130–139, 140–159, 160–179, and ≥180 mmHg), DBP group (< 80, 80–89, 90–99, 100–109, and ≥110 mmHg), BMI group (normal, overweight, and obese), smoking status (never smoked, ever smoked, and current smoker), and drinking status (never consumed alcohol, ever consumed alcohol, and currently consumes alcohol). Cox proportional hazards models were performed to estimate the risk of incident stroke by BP category using models adjusted for age, sex, education level, BMI, smoking status, and drinking status. The follow-up time (recorded in years) was calculated as the interval between the date at baseline and the date of the occurring stroke for patients experiencing a first-ever stroke during the study period. For participants without further stroke events, the follow-up time was defined as 26.3 years. Moreover, for participants who died during the study periods, the follow-up time was defined as the interval between baseline and the date of death. Data for patients who were lost to follow-up or who emigrating were censored. The adjusted hazard ratios (HRs) for the incidence of overall stroke and each subtype were presented the association of BP levels with the stroke risk.

For except for the interaction of age, BP, and stroke types on the stroke risk, we evaluated the interaction of BP, age, and stroke subtype for stroke risk using the Cox regression model, with HR (95%CI) of 1.021 (1.020, 1.023; *P* = 0.021). Further, a subgroup analysis was performed to detect the association of BP with stroke risk, by age (simplified to < 65 years and ≥65 years), because there was the interaction among age, BP, and stroke types.

Diabetes and physical activity were not analyzed in this study because there were few patients with diabetes (*n* = 4), at baseline, and none had baseline physical activity measurements. All statistical analyses were performed using SPSS for Windows (version 15.0; SPSS, Chicago, IL, USA); a *P*-value < 0.05 was considered statistically significant.

## Results

**Figure 1:**
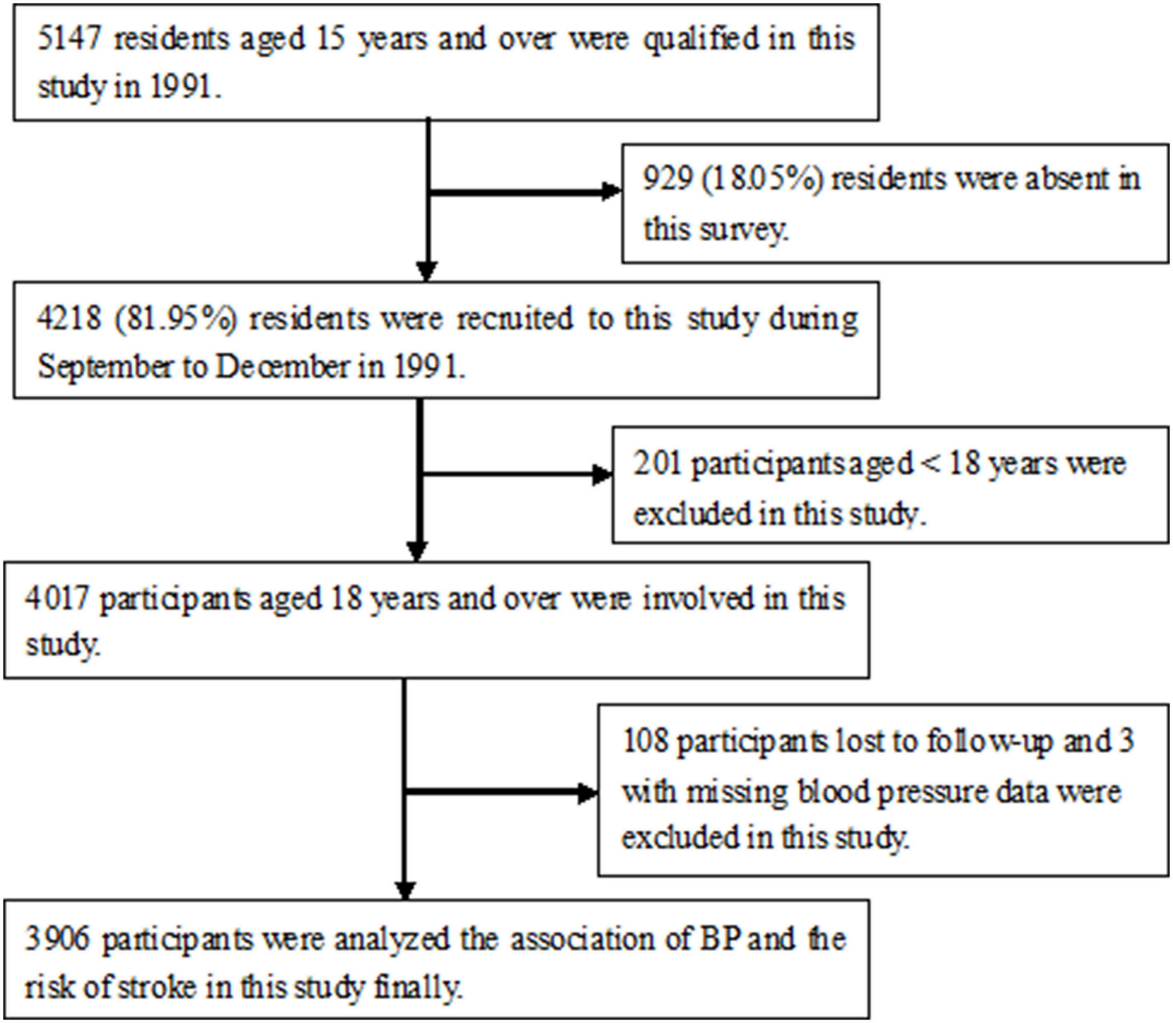
Flow chart showed that 4,218 were recruited into this survey (response rate = 82%), among 5,147 individuals aged ≥15-years-old. Of these, 4,017 individuals were ultimately enrolled, after excluding 201 individuals aged < 18 years. During the course of the study, 108 participants were lost to follow-up, and three with missing baseline BP data were removed from the BP analysis. Finally, a total of 3,906 participants were analyzed in this study.

Overall, 5,147 individuals were ≥15-years-old, and 4,218 were recruited into this survey (response rate = 82%). Of these, 4,017 individuals were ultimately enrolled, after excluding 201 individuals aged < 18 years. During the course of the study, 108 participants were lost to follow-up, and three with missing baseline BP data were removed from the BP analysis. Finally, a total of 3,906 participants were evaluated to determine the association between BP and the incidence of first-ever strokes (Figure 1).

### Demographic Features and Distribution of CVD Risk Factors at Baseline

Of the 4017 participants included in this study, 1841 (45.8%) were men and 2176 (54.2%) were women. This group represented a total of 85424.6 person-years of follow-up, with a mean follow-up period of 26.28 years. At baseline, the average participant age was 41.50 years; >80% were < 65-years-old. A large proportion (40.7%) of participants had received no formal education. At baseline, 22.0% of the participants had SBPs ≥140 mmHg, and 25.9% had DBPs ≥90 mmHg. The baseline frequencies of overweight and obesity were 21.5 and 4.2%, respectively; the baseline frequencies of current smoking and drinking were 25.0 and 15.0%, respectively (Table 1).

**Table 1:** Description of the demographical features among at baseline all participants by BP.

**Features**	** < 65 years**	**≥ 65 years**	**Total**
Participants	3,527 (87.8)	490 (12.2)	4,017 (100)
Men	1,588 (45.0)	253 (51.6)	1,841 (45.8)
Follow-up time, years	26.28 (0.90)	7.75 (11.01)	26.28 (7.29)
Person-year	80707.17	4717.43	85424.60
Age, years	37.21 (12.59)	72.37 (6.09)	41.50 (16.62)
**EDUCATION ATTAINMENT**			
0 year	1,220 (34.6)	415 (84.7)	1,635 (40.7)
1~6 years	926 (26.3)	68 (13.9)	994 (24.7)
7~9 years	1,240 (35.2)	5 (1.0)	1,245 (31.0)
≥ 10 years	141 (4.0)	2 (0.4)	143 (3.6)
SBP, mmHg	124.08 (16.60)	149.76 (28.06)	127.22 (20.21)
**SBP GROUPS**			
< 130	2,226 (63.1)	80 (16.3)	2,306 (57.4)
130~	742 (21.0)	85 (17.3)	827 (20.6)
140~	396 (11.2)	165 (33.7)	561 (14.0)
160~	100 (2.7)	75 (15.3)	175 (4.4)
≥180	63 (1.8)	85 (17.3)	148 (3.7)
DBP, mmHg	78.80 (10.51)	87.88 (13.86)	79.91 (11.37)
**DBP GROUPS**			
< 80	1,327 (37.6)	84 (17.1)	1,411 (35.1)
80~	1,442 (40.9)	123 (25.1)	1,565 (39.0)
90~	586 (16.6)	174 (35.5)	760 (18.9)
100~	122 (3.5)	62 (12.7)	184 (4.6)
≥110	50 (1.4)	47 (9.6)	97 (2.4)
BMI, Kg/m^2^	22.69 (2.76)	21.83 (2.96)	22.58 (2.80)
**BMI GROUPS**			
Normal	2,595 (73.6)	391 (79.8)	2,986 (74.3)
Over weight	779 (22.1)	95 (17.3)	864 (21.5)
Obesity	153 (4.3)	14 (2.9)	167 (4.2)
**SMOKING STATUS**			
Never	2,530 (71.7)	369 (75.3)	2,899 (72.2)
Ever	96 (2.7)	16 (3.3)	112 (2.8)
Current	901 (25.5)	105 (21.4)	1,006 (25.0)
**DRINKING STATUS**			
Never	2,952 (83.7)	445 (90.8)	3,397 (84.6)
Ever	16 (0.5)	0	16 (0.4)
Current	559 (15.8)	45 (9.2)	604 (15.0)

*SD, standard deviation; SBP, systolic blood pressure; DBP, diastolic blood pressure; BMI, body mass index*.

### Age-Standardized Incidence of First-Ever Stroke/1,000 Person-Years, by Stroke Type

Over the 27-year follow-up period, 16.3% of participants (638 participants; 259 women) experienced first-ever strokes. Of these, 19.0% (121 participants; 41 women) experienced hemorrhagic strokes.

Table 2 shows that the age-standardized incidence of first-ever stroke/1,000 person-years was 10.1, overall, including 5.8 for ischemic stroke and 1.8 for hemorrhagic stroke. The overall incidence of stroke was higher for men (12.8) than for women (7.7); this pattern existed for both ischemic (5.8 vs. 4.7) and hemorrhagic (2.5 vs. 1.2) strokes (all, *P* < 0.001).

**Table 2:** Age-standardized incidence incidences of first-ever stroke in this cohort study during 27-years following–up periods by stroke subtypes (per 1,000 person-year).

**Features**	**Age-standardized incidence (95%CI)**			***P***
	**Stroke**	**Ischemic stroke**	**Hemorrhagic stroke**	
GENDER^*^				
Men	12.8 (11.7, 13.9)	5.8 (5.0, 6.6)	2.5. (2.0., 3.0.)	0.001
Women	7.7 (6.9, 8.5)	4.7 (4.0, 5.3)	1.2 (0.9, 1.5)	0.045
Total	10.1 (9.5, 20.8)	5.8 (5.3, 6.2)	1.8 (1.5, 2.1)	< 0.001
*P* for trend	< 0.001	< 0.001	< 0.001	
**AGE GROUPS, YEARS**				
18~34	2.2 (1.7, 2.6)	1.6 (1.8, 2.0)	0.6 (0.4, 0.8)	0.622
35~44	5.5 (4.5, 6.5)	4.3 (3.4, 5.6)	1.0 (0.5, 1.4)	0.156
45~54	11.1 (9.0, 13.1)	7.8 (6.1, 9.5)	2.3 (1.3, 3.2)	0.020
55~64	20.1(17.1, 23.2)	13.4 (10.9, 15.9)	3.5 (2.2, 4.8)	0.003
65~74	32.7 (27.0, 38.4)	13.1 (9.5, 16.8)	5.6 (3.2, 8.0)	0.054
≥75	33.5 (22.3, 44.8)	9.1 (3.2, 15.1.)	3.0 (4.0, 6.5)	1.000
*P* for trend	< 0.001	< 0.001	< 0.001	
EDUCATION ATTAINMENT^*^, Years				
0	10.3 (9.1, 11.4)	5.6 (4.8, 6.5)	2.6 (2.0, 3.2)	0.002
1~6	8.5 (7.3, 9.7)	6.0 (5.0, 7.0)	2.0 (1.4, 2.5)	0.013
7~9	9.1 (8.1, 10.2)	5.8 (4.5, 6.7)	0.7 (0.4, 1.0)	1.000
≥10	1.2 (0, 2.3)	1.0 (0.1, 1.8)	0.3 (0.3, 0.9)	1.000
*P* for trend	< 0.001	< 0.001	< 0.001	
SBP GROUPS^*^, mmHg				
< 130	7.9 (7.2, 8.7)	5.2 (4.6, 5.8)	1.4 (1.1, 1.7)	0.047
130~	8.8 (7.4, 10.2)	5.3 (4.3, 6.4)	1.5 (9.4, 2.0)	0.100
140~	11.8 (9.6, 13.9)	7.1 (5.5, 8.8)	2.1 (1.1, 2.9)	0.022
160~	20.3 (14.6, 26.1)	10.7 (6.6, 15.1)	6.4 (3.2, 9.8)	0.043
≥180	19.7 (13.0, 26.9)	8.9 (4.3, 13.7)	4.6 (1.2, 7.8)	0.355
*P* for trend	< 0.001	< 0.001	< 0.001	
DBP GROUPS^*^, mmHg				
< 80	7.1 (6.2, 8.0)	4.8 (4.1, 5.6)	1.2 (0.8, 1.5)	0.253
80~	8.6 (7.6, 9.6)	4.8 (4.1, 5.5)	1.6 (1.2, 2.2)	0.028
90~	11.7 (10.1, 13.7)	7.1 (5.7, 8.5)	1.8 (1.1, 2.6)	0.015
100~	16.0 (11.2, 20.7)	9.3 (5.2, 12.9)	3.3 (1.2, 5.5)	0.127
≥110	36.0 (25.3, 47.1)	16.3 (9.3, 24.3)	1.3 (0.8, 2.6)	0.113
*P* for trend	< 0.001	< 0.001	< 0.001	
BMI GROUPS^*^, m^**2**^**/Kg**				
Normal	9.3 (8.69, 10.1)	5.3 (4.7, 5.8)	1.5 (1.2, 1.8)	0.007
Over weight	12.6 (10.1, 13.9)	6.9 (5.7, 8.1)	2.6 (1.9, 3.4)	0.007
Obesity	11.7 (8.0, 15.4)	8.0 (4.9, 10.1)	1.5 (0.1, 2.9)	0.585
*P* for trend	< 0.001	< 0.001	0.001	
SMOKING STATUS^*^				
Never	9.3 (8.6, 10.1)	5.3 (4.7, 5.9)	1.8 (1.5, 2.2)	0.006
Ever	12.6 (8.0, 17.4)	10.0 (5.8, 14.1)	1.5 (0.2, 2.9)	0.325
Current	12.3 (10.9, 13.8)	6.6 (5.5, 7.6)	2.4 (1.8, 3.0)	0.013
*P* for trend	< 0.001	< 0.001	0.013	
DRINKING STATUS^*^				
Never	10.1 (9.4, 10.8)	5.3 (4.8, 5.8)	1.8 (1.5, 2.1)	0.001
Ever	23.6 (7.7, 41.4)	16.8 (3.7, 33.1)	6.8 (2.4, 14.7)	0.475
Current	11.9 (10.1, 13.7)	5.7 (4.4, 6.9)	2.2 (1.4, 2.9)	0.077
*P* for trend	0.003	0.001	0.778	

* *presented as age-standardized incidence using WHO standardized population; 95%CI, 95% confidence interval; SBP, systolic blood pressure; DBP, diastolic blood pressure; BMI, body mass index*.

Furthermore, the age-standardized incidence of first-ever stroke associated with age, educational attainment, SBP and DBP levels, BMI groups, smoking status, and drinking status (all, *P* < 0.05) in the univariate analysis.

### Association of SBP and DBP With First-Ever Stroke Risk in Men and Women, by Stroke Type

The stroke risk was 58% higher among individuals with SBP values of 140–159 mmHg than among those with SBP values of < 130 mmHg (the reference group), after adjusting for age, sex, educational level, BMI, smoking status, and drinking status (all *P* < 0.05). Accordingly, the stroke risk increased 1.56-fold among those with SBP values of 160–179 mmHg and 2.08-fold among those with SBP values ≥180 mmHg, compared with the reference group (both, *P* < 0.05). Similarly, the ischemic stroke risk increased by 60%, 1.67-fold, and 1.4-fold, in the respective groups. However, hemorrhagic stroke risk increased only among participants with SBPs ≥160 mmHg, with increases of 2.3-fold among those with SBP values of 160–179 mmHg and 2.12-fold among those with SBPs ≥180 mmHg.

Simultaneously, stroke risk increased significantly among participants with DBP values of ≥90 mmHg compared to the reference group of individuals (DBP < 80 mmHg); both in IS and in ICH (Table 3).

**Table 3:** Adjusted hazard ratio of BP levels for the incidence of the first-ever stroke in this cohort study by stroke types (95% CI).

**BP category**	**Stroke**	**IS**	**ICH**
**TOTAL**			
SBP, mmHg	1.016 (1.012, 1.019)^*^	1.013 (1.008, 1.018)^*^	1.020 (1.011, 1.029)^*^
< 130	1.00	1.00	1.00
130~	1.11 (0.89, 1.38)	1.14 (0.88, 1.48)	0.98 (0.59, 1.61)
140~	1.58 (1.26, 1.99)^*^	1.60 (1.20, 2.12)^*^	1.44 (0.84, 2.44)
160~	2.56 (1.90, 3.46)^*^	2.67 (1.81, 3.94)^*^	3.30 (1.70, 6.42)^*^
≥180	3.08 (2.23, 4.26)^*^	2.40 (1.50, 3.84)^*^	3.12 (1.41, 6.87)^*^
DBP, mmHg	1.030 (1.023, 1.037)^*^	1.027 (1.018, 1.036)^*^	1.038 (1.022, 1.055)^*^
< 80	1.00	1.00	1.00
80~	1.18 (0.95, 1.46)	1.03 (0.80, 1.34)	1.57 (0.95, 2.58)
90~	1.64 (1.31, 2.05)^*^	1.55 (1.17, 2.04)^*^	1.79 (1.03, 3.12)^*^
100~	2.36 (1.72, 3.22)^*^	2.16 (1.45, 3.24)^*^	3.23 (1.56, 6.67)^*^
≥110	4.51 (3.16, 6.43)^*^	3.89 (2.40, 6.29)^*^	6.41 (2.85, 14.41)^*^
**MEN**			
SBP, mmHg	1.019 (1.014, 1.025)^*^	1.017 (1.010, 1.025)^*^	1.021 (1.009, 1.033)^*^
< 130	1.00	1.00	1.00
130~	1.17 (0.89, 1.53)	1.15 (0.82, 1.61)	1.19 (0.68, 2.10)
140~	1.59 (1.18, 2.14)^*^	1.78 (1.23, 2.59)^*^	1.12 (0.56, 2.26)
160~	3.02 (2.01, 4.56)^*^	2.75 (1.54, 4.89)^*^	4.24 (1.88, 9.55)^*^
≥180	3.01 (1.90, 4.79)^*^	2.78 (1.42, 5.42)^*^	2.26 (0.74, 6.97)
DBP, mmHg	1.037 (1.028, 1.047)^*^	1.034 (1.022, 1.047)^*^	1.045 (1.025, 1.066)^*^
< 80	1.00	1.00	1.00
80~	1.39 (1.04, 1.84)^*^	1.24 (0.87, 1.76)	1.85 (0.98, 3.48)
90~	1.91 (1.42, 2.58)^*^	1.95 (1.35, 2.83)^*^	1.88 (0.93, 3.82)
100~	2.01 (1.27, 3.17)^*^	2.09 (1.17, 3.75)^*^	3.16 (1.22, 8.22)^*^
≥ 110	5.33 (3.30, 8.61)^*^	4.22 (2.10, 8.51)^*^	7.29 (2.66, 19.96)^*^
**WOMEN**			
SBP, mmHg	1.012 (1.007, 1.018)^*^	1.010 (1.003, 1.017)^*^	1.019 (1.005, 1.032)^*^
< 130	1.00	1.00	1.00
130~	1.03 (0.72, 1.50)	1.22 (0.81, 1.86)	0.44 (0.13, 1.53)
140~	1.48 (1.04, 2.11)^*^	1.29 (0.82, 2.02)	1.92 (0.84, 4.38)
160~	2.00 (1.28, 3.11)^*^	2.31 (1.36, 3.92)^*^	1.86 (0.57, 6.08)
≥180	2.83 (1.79, 4.46)^*^	2.00 (1.04, 3.87)^*^	3.28 (1.06, 10.14)^*^
DBP, mmHg	1.023 (1.013, 1.033)^*^	1.020 (1.007, 1.033)^*^	1.027 (1.001, 1.053)^*^
< 80	1.00	1.00	1.00
80~	1.02 (0.73, 1.42)	0.92 (0.62, 1.36)	1.11 (0.49, 2.53)
90~	1.27 (0.89, 1.80)	1.18 (0.77, 1.80)	1.38 (0.56, 3.37)
100~	2.68 (1.74, 4.13)^*^	2.31 (1.33, 4.03)^*^	2.52 (0.82, 7.68)
≥110	3.45 (2.05, 5.81)^*^	3.39 (1.76, 6.21)^*^	3.53 (0.91, 13.61)

* *indicated P < 0.05*.

Further, we assessed the SBP and DBP as a continuous variable in Cox proportional hazard model. Each 1 mmHg increasement of SBP resulted in 1.6% increased risk of stroke, 1.3% for IS and 2.0% for ICH. The corresponding value of DBP was 3.0% overall, 2.7% for IS and 3.8% for ICH. Similar trends were found both in men and in women (Table 3).

### Association of SBP and DBP With First-Ever Stroke Risk, by Age, Sex, and Stroke Types

Among individuals aged < 65 years, stroke risk increased as SBP and DBP increased, both in IS and in ICH. The stroke risk increased in those with SBP of ≥140 mmHg across sex and stroke types, except for men with ≥160 mmHg for ICH. Given DBP, The stroke risk increased in women with DBP of ≥100 mmHg across stroke types, but lower DBP level was found in men.

However, among individuals aged 65 years and old, the stroke risk increased in those with SBP of ≥180mmHg both in men and in women for stroke; the stroke risk increased in men with DBP of ≥100 mmHg and in women ≥90 mmHg.

Moreover, the quantitative analysis showed that the stroke risk increased significantly with advanced BP levels across sex and stroke types among individuals aged < 65 years old. However, the stroke risk increased with DBP level for IS in women aged ≥65 years old and for ICH in men aged ≥65 years old (Table 4).

**Table 4:** Adjusted hazard ratio of BP levels for the incidence of the first-ever stroke by age, sex, and stroke types in this cohort study (95% CI).

**Groups**	**Stroke**		**IS**		**ICH**	
	** < 65 years**	**≥ 65 years**	** < 65 years**	**≥ 65 years**	** < 65 years**	**≥ 65 years**
**TOTAL**						
SBP, mmHg	1.016 (1.012, 1.021)^*^	1.014 (1.008, 1.020)^*^	1.015 (1.009, 1.021)^*^	1.009 (0.999, 1.019)	1.023 (1.012, 1.034)^*^	1.012 (0.996, 1.029)
< 130	1.00	1.00	1.00	1.00	1.00	1.00
130~	1.37 (1.09, 1.73)^*^	1.16 (0.64, 2.11)	1.44 (1.10, 1.89)^*^	0.80 (0.32, 1.99)	1.14 (0.68, 1.93)	0.86 (0.21, 3.50)
140~	2.33 (1.81, 3.01)^*^	1.65 (0.98, 2.78)	2.44 (1.81, 3.28)^*^	1.11 (0.52, 2.40)	2.17 (1.24, 3.80)^*^	0.86 (0.24, 3.11)
160~	5.36 (3.75, 7.64)^*^	2.19 (1.21, 3.97)^*^	5.15 (2.34, 7.94)^*^	1.62 (0.68, 3.87)	4.53 (1.98, 10.37)^*^	2.82 (0.80, 9.88)
≥180	5.47 (3.52, 8.49)^*^	3.35 (1.86, 6.06)^*^	4.61 (2.62, 8.08)^*^	1.51 (0.57, 3.97)	6.28 (2.54, 15.52)^*^	1.72 (0.36, 8.20)
DBP, mmHg	1.029 (1.020, 1.037)^*^	1.033 (1.021, 1.046)^*^	1.026 (1.016, 1.037)^*^	1.025 (1.005, 1.046)^*^	1.040 (1.021, 1.059)^*^	1.031 (1.000, 1.063)
< 80	1.00	1.00	1.00	1.00	1.00	1.00
80~	1.26 (1.00, 1.59)^*^	1.43 (0.82, 2.50)	1.16 (0.88, 1.52)	0.73 (0.31, 1.72)	1.97 (1.15, 3.35)^*^	0.56 (0.12, 2.57)
90~	2.15 (1.67, 2.78)^*^	2.06 (1.22, 3.46)^*^	2.20 (1.64, 2.96)^*^	1.18 (0.55, 2.54)	2.09 (1.11, 3.91)^*^	1.78 (0.55, 5.79)
100~	3.69 (2.54, 5.38)^*^	2.65 (1.42, 4.65)^*^	3.04 (1.90, 4.87)^*^	2.19 (0.92, 5.20)	4.65 (2.00, 10.82)^*^	2.22 (0.53, 9.36)
≥110	6.60 (4.20, 10.37)^*^	4.70 (2.45, 9.00)^*^	6.22 (3.60, 10.77)^*^	2.33 (0.82, 6.63)	9.22 (3.58, 23.71)^*^	3.61 (0.75, 17.46)
**MEN**						
SBP, mmHg	1.035 (1.027, 1.042)^*^	1.017 (1.008, 1.025)^*^	1.034 (1.025, 1.043)^*^	1.008 (0.994, 1.023)	1.034 (1.019, 1.049)^*^	1.015 (0.995, 1.035)
< 130	1.00	1.00	1.00	1.00	1.00	1.00
130~	1.19 (0.89, 1.60)	1.00 (0.49, 2.02)	1.25 (0.88, 1.77)	0.37 (0.11, 1.26)	1.18 (0.64, 2.18)	1.04 (0.23, 4.74)
140~	1.69 (1.19, 2.37)^*^	1.36 (0.73, 2.54)	1.97 (1.32, 2.95)^*^	0.85 (0.35, 2.06)	1.32 (0.61, 2.86)	0.69 (0.15, 3.12)
160~	3.95 (2.36, 6.61)^*^	2.06 (0.99, 4.30)	3.64 (1.90, 7.00)^*^	0.95 (0.28, 3.18)	4.47 (1.57, 12.71)^*^	3.08 (0.72, 13.21)
≥180	2.42 (1.23, 4.75)^*^	3.11 (1.45, 3.08)^*^	2.81 (1.25, 6.33)^*^	1.19 (0.33, 4.26)	2.19 (0.48, 9.94)	2.12 (0.32, 13.99)
DBP, mmHg	1.052 (1.040, 1.063)^*^	1.037 (1.020, 1.054)^*^	1.052 (1.038, 1.065)^*^	1.018 (0.990, 1.047)	1.054 (1.030, 1.078)^*^	1.047 (1.009, 1.087)^*^
< 80	1.00	1.00	1.00	1.00	1.00	1.00
80~	1.49 (1.09, 2.04)^*^	1.50 (0.76, 2.95)	1.44 (0.98, 2.10)	0.65 (0.23, 1.80)	2.39 (1.19, 4.79)^*^	0.56 (0.09, 3.37)
90~	2.76 (1.97, 3.88)^*^	1.82 (0.96, 3.43)	3.01 (2.01, 4.49)^*^	0.93 (0.38, 2.29)	2.46 (1.09, 5.55)^*^	1.37 (0.34, 5.51)
100~	3.31 (1.86, 5.91)^*^	2.38 (1.06, 5.34)^*^	3.62 (1.83, 7.14)^*^	1.68 (0.52, 5.39)	3.23 (0.81, 11.99)	3.61 (0.76, 17.10)
≥110	7.37 (3.83, 14.18)^*^	5.32 (2.37, 11.93)^*^	7.15 (3.17, 16.15)^*^	1.54 (0.38, 6.26)	9.78 (2.65, 36.18)^*^	6.74 (1.22, 37.09)^*^
**WOMEN**						
SBP, mmHg	1.025 (1.019, 1.032)^*^	1.009 (1.001, 1.018)^*^	1.023 (1.015, 1.031)^*^	1.008 (0.995, 1.021)	1.029 (1.014, 1.043)^*^	1.007 (0.980, 1.035)
< 130	1.00	1.00	1.00	1.00	1.00	1.00
130~	1.20 (0.81, 1.77)	1.70 (0.56, 5.19)	1.34 (0.87, 2.08)	3.47 (0.65, 18.66)	0.55 (0.16, 1.88)	—
140~	2.02 (1.36, 2.99)^*^	1.96 (0.76, 5.07)	1.94 (1.21, 3.09)^*^	1.77 (1.34, 9.28)^*^	2.38 (1.02, 5.54)^*^	1.15 (0.10, 13.85)
160~	3.81 (2.29, 6.31)^*^	2.62 (0.96, 7.14)	3.98 (2.21, 7.18)^*^	5.12 (1.05, 25.02)^*^	2.00 (0.45, 8.85)	2.20 (0.19, 25.45)
≥180	5.18 (2.94, 9.13)^*^	3.25 (1.24, 8.48)^*^	4.19 (1.98, 8.90)^*^	2.58 (0.46, 14.34)	5.95 (1.88, 18.88)^*^	1.15 (2.07, 19.09)
DBP, mmHg	1.035 (1.023, 1.047)^*^	1.027 (1.009, 1.045)^*^	1.031 (1.016, 1.045)^*^	1.033 (1.005, 1.061)^*^	1.043 (1.014, 1.072)^*^	0.997 (0.939, 1.058)
< 80	1.00	1.00	1.00	1.00	1.00	1.00
80~	1.02 (0.72, 1.45)	1.21 (0.45, 3.24)	0.94 (0.63, 1.41)	1.18 (0.25, 5.48)	1.19 (0.51, 2.82)	0.64 (0.04, 10.50)
90~	1.47 (0.99, 2.19)	2.43 (1.01, 5.83)^*^	1.51 (0.96, 2.37)	1.93 (0.48, 7.78)	1.28 (0.46, 3.61)	2.20 (0.31, 25.67)
100~	3.81 (2.29, 6.31)^*^	3.28 (1.23, 8.78)^*^	2.78 (1.45, 5.30)^*^	5.10 (1.22, 21.29)^*^	4.28 (1.40, 13.11)^*^	-
≥110	5.18 (2.94, 9.13)^*^	3.17 (1.08, 9.29)^*^	4.89 (2.34, 10.20)^*^	3.47 (0.67, 18.02)	6.08 (1.71, 26.12)^*^	-

* *indicated P < 0.05*.

## Discussion

In this 27-year prospective cohort study of a low-income population, in China, we assessed the stroke risk associated with different BP levels, according to age, sex, and stroke type. The overall stroke risk increased with increasing SBP and DBP levels, regardless of patient sex or stroke type. Stroke risk increased among individuals with SBP levels ≥140 mmHg (for ischemic stroke) and ≥160 mmHg (for ICH), compared with those with SBP levels < 130 mmHg. However, the stroke risk increased among those with DBP levels ≥ 90 mmHg, for both ischemic stroke and ICH, compared with individuals with DBP levels < 80 mmHg. Specifically, in men, the stroke risk increased among those with SBP levels ≥140 mmHg (ischemic stroke) or ≥160 mmHg (ICH) and among those with DBP levels ≥90 mmHg both for ischemic stroke and ICH. However, in women, the stroke risk increased among those with SBP levels ≥160 mmHg (ischemic stroke) or ≥180 mmHg (ICH) and among those with DBP levels ≥100 mmHg (ischemic stroke only).

Over the past few decades, the stroke incidence has been decreasing in developed countries (23–26) but increasing in developing countries, especially in China (8, 27, 28). A study from Japan demonstrated a lower incidence of stroke than we found, in this study, with 213 stroke events occurring during a follow-up period of 64,395 person-years among individuals 30–79-years-old (29). The Circulatory Risk in Communities Study reported the age- and sex-adjusted stroke incidences, per 1,000 person-years, for three cohorts, as: 4.6 for the 1963–1971 cohort, 3.6 for the 1975–1984 cohort, and 2.7 for the 1985–1994 cohort (30). The Framingham Study documented a much lower first-ever stroke incidence, during a 51-year follow-up period (115,146 person-years) (15). In the present study, the age-adjusted incidence of first-ever stroke was 10.1/1,000 person-years. The greater stroke incidence in our study may have been associated with the higher prevalence of related risk factors (4).

Several studies have demonstrated a positive association between BP and CVD risk, based on long-term observations. Based on 30 years of observations, the Framingham study reported that the lifetime risk of first-ever stroke increased as BP levels increased, in both men and women at 55 years of age (15). A 28-year follow-up study from Göteborg, Sweden, reported a significant association between SBP levels and stroke events in men aged 47–55 years (31). A similar association was found in the Hisayama study, conducted in a cohort of Japanese individuals ≥40-years-old (30). Additionally, a significant association between BP level and mortality due to stroke was reported after a 24-year observation of Japanese individuals 30–92-years-old (32).

Epidemiological studies have demonstrated that elevated SBP and DBP levels are both associated with increased CVD risk (33, 34). Higher SBP levels are consistently associated with increased CVD risk, after adjusting or stratification for DBP (35–37). In contrast, another study reported that DBP was not consistently associated with CVD risk, after considering the SBP levels following adjustment or stratification (38, 39). Consistent with the results of that study, we found a positive association between DBP levels and stroke risk for both ischemic and hemorrhagic stroke in this low-income population, in China. A >5-fold increase in hemorrhagic stroke risk was observed among participants with DBP levels ≥110 mmHg.

In another analysis, a 20-mmHg increase in SBP and a 10-mmHg increase in DBP were each associated with a doubling of the risk of death due to stroke, heart disease, or other vascular disease (13). In a separate observational study that included >1 million adult patients (≥30 years of age), elevated SBP and DBP values were associated with increased risks of CVD, including angina, myocardial infarction, heart failure, stroke, peripheral artery disease, and abdominal aortic aneurysm, each evaluated separately (14). In this study, compared to the reference group (SBP < 130 mmHg or DBP < 80 mmHg), the overall and ischemic stroke risks were significantly increased among those with SBP values ≥140 mmHg or DBP values ≥90 mmHg, after adjusting for conventional CVD risk factors. However, the hemorrhagic stroke risk increased among those with SBP values ≥160 mmHg and/or DBP values ≥90 mmHg. In this study, the ideal BP cut-off values for decreasing stroke risk were SBP values ≤ 140 mmHg and DBP values ≤ 90 mmHg, for overall and ischemic stroke; for hemorrhagic stroke, the cut-off values were ≤ 160 mmHg for SBP and ≤ 90 mmHg for DBP.

Among older Japanese adults with isolated systolic hypertension and baseline SBP values ≥160 mm Hg, the on-treatment SBP level at which CVD event risks and all-cause mortality were minimized was 130 to < 145 mmHg. On-treatment SBP values of < 130 or ≥145 mmHg were associated with increased CVD event risk and all-cause mortality (40). Thus, decreasing SBP values to < 130 mmHg in individuals ≥60-years-old with isolated systolic hypertension can result in adverse cardiovascular outcomes (41, 42). The Joint National Committee 8 panel recommended 150 mmHg as the treatment target for older adults (43). In the present study, we found a disparity in the threshold BP value predicting first-ever stroke risk. The target BP value for reducing stroke risk was < 130 mmHg for SBP and < 90 mmHg for DBP among individuals < 65-years-old. However, among individuals ≥65-years-old, the values were < 160 mmHg for SBP and < 90 mmHg for DBP. Moreover, the threshold SBP/DBP value was ≥130/90 mmHg for ischemic stroke and ≥140/80 mmHg for hemorrhagic stroke among individuals aged < 65 years. However, there was no significant association between the SBP/DBP value and the risk of specific stroke subtypes (including ischemic and hemorrhagic stroke) among the elderly.

This study has several limitations. First, the study population was from a township in northern China, which is not representative of China's overall population. However, the study's prospective design and long duration may have reduced the impact of the limited generalizability of the results. Second, the total of 85,000 person-years in the follow-up period did not fulfill the minimum criterion of 100,000 person-years for population studies (44). Third, diabetes, using medicine, and physical activity were not adjusted in Cox regression analysis; these lacking information may impact the assessment of association between stroke risk and risk factors. However, the population in this study is a low-income, low-education, which is from a township in Tianjin, China. In this study, the history of diabetes was self-reported, thus, there were only four participants with known diabetes among those individuals without previous stroke at baseline. In 1992, we selected 1,092 individuals aged 35–64 years among this cohort to measure the level of FBG. The prevalence of diabetes was 1.9% according to FBG ≥ 7.0mmol/L, but the awareness rate was 42.9%. Moreover, medical and medication history as well as physical activity data were only available at baseline. The change in the last three decades would have huge impact on the outcome, However, it was impossible to consider in the multivaraible analyses. In this study, none of the patients with the known history of hypertension or diabetes received standardized treatment in this population at baseline. Except for physical labor, there is not any physical activity in this population at baseline. Thus, further study will be needed. Finally, we did not collect detailed information regarding dietary habits and blood examination at baseline; therefore, other possible determinants of stroke could not be assessed.

## Conclusions

Overall, the findings from this 27-year, prospective cohort study suggest the critical need to monitor and manage both SBP and DBP to reduce the stroke risk among low-income Chinese individuals. Moreover, a lower threshold BP value for predicting first-ever stroke was observed for middle-aged adults than for the elderly, in this population. To reduce the stroke burden in China, BP management goals must address middle-aged adults and provide different BP targets for middle-aged and elderly adults. This finding is very important for guiding clinical practice; moreover, it may generalize to other developing countries experiencing rapid economic development and transitions in the spectrum of disease.

## Data Availability

All datasets generated for this study are included in the manuscript and/or the supplementary files.

## Ethics Statement

This study was carried out in accordance with the recommendations of the ethics committee of Tianjin Medical University General Hospital with written informed consent from all subjects. All subjects gave written informed consent in accordance with the Declaration of Helsinki. The protocol was approved by the ethics committee of Tianjin Medical University General Hospital.

## Author Contributions

XN, QY, and JW contributed to the conception and design of the work. XD, CW, JN, HG, JL, JP, and JT contributed the data acquisition. JW and XN contributed the analysis and interpretation of data for the work. XD and CW contributed drafting the work. XN, QY, and JW contributed revising the work for important intellectual content. All authors approved of the final version to be published, and agree to be accountable for all aspects of the work in ensuring that questions related to the accuracy or integrity of any part of the work are appropriately investigated and resolved.

### Conflict of Interest Statement

The authors declare that the research was conducted in the absence of any commercial or financial relationships that could be construed as a potential conflict of interest.
